# The gender gap in adolescents’ emotional and behavioural problems in Georgia: a cross-sectional study using Achenbach’s Youth Self Report

**DOI:** 10.1186/s13034-023-00592-0

**Published:** 2023-03-30

**Authors:** Khatia Antia, Justina Račaitė, Genė Šurkienė, Volker Winkler

**Affiliations:** 1 Heidelberg Institute of Global Health, University Hospital Heidelberg, Im Neuenheimer Feld 130.3, 69120 Heidelberg, Germany; 2 School of Health Sciences, The University of Georgia, 77a Kostava Street, Tbilisi, 0175 Georgia; 3 Department of Public Health, Institute of Health Sciences, Faculty of Medicine, Vilnius University, M. K. Čiurlionio str. 21, Vilnius, LT-03101 Lithuania

**Keywords:** Adolescents, Gender gap, Mental health, Emotional difficulties, Problem behaviour, Self-report, Left-behind, Georgia

## Abstract

**Background:**

Adolescents, particularly girls, are vulnerable to mental health disorders. Knowledge about young people’s mental health in Eastern European countries is limited. This study is the first to investigate adolescents’ self-reported emotional and behavioural problems in Georgia from a public mental health perspective.

**Methods:**

This study utilized Achenbach’s Youth Self-Reported syndrome scales among 933 adolescents studying from grades 7–12 in 18 public schools in Georgia. We compared the gender-specific results with each other and with the Achenbach’s Normative Sample using two-sample t-tests. Linear regression was used to assess associations between internalizing and externalizing problems and individual as well as demographic characteristics such as parental migration experience (being ‘left-behind’ or ‘staying behind’).

**Results:**

The study found that girls obtained higher scores than boys in Youth Self-Reported empirical syndrome scales and the internalizing broadband scale. Rule-breaking behaviour was the only syndrome scale where boys scored higher. Compared to Achenbach’s Normative Sample, adolescents in Georgia scored higher on all scales. Regression analyses showed that illnesses, having fewer than three close friends, problems at school, and worse relationships with peers/siblings/parents (compared to peers) were associated with higher internalizing and externalizing problems scores in both genders. Performing household chores, living with only one parent, or having a migrant parent showed no associations in either gender.

**Conclusion:**

Emotional and behavioural difficulties of adolescents in Georgia, especially girls, require attention. Having close friends, developing strong relationships with family members, and a supportive school environment could help mitigate emotional and behavioural problems among adolescents in Georgia.

**Supplementary Information:**

The online version contains supplementary material available at 10.1186/s13034-023-00592-0.

## Background

Adolescence is a transitional period into adulthood, a unique time essential for human development [1, 2]. Increased stress, anxiety, and behavioural changes make this period challenging for youth [2, 3]. Available literature suggests that around 50% of lifetime mental illnesses develop during the teenage years [4]. Adolescents with poor mental health may experience personal, academic and social challenges [5].

Various outcome measures are applied to examine the mental health of children and adolescents [6], among which internalizing and externalizing are the most commonly used broadband self-reported measures [7]. When investigating internalizing and externalizing problems, scholars observe a universal gender gap, specifically, worse internalizing outcomes among girls and worse externalizing outcomes among boys [8–14]. Moreover, the risk of developing depression and anxiety is twice as high in adolescent girls as in boys [7, 15–17]. Evidence from European studies suggest that older adolescents are worse off than younger ones [8–14].

Georgia is an Eastern European, post-Soviet country and one of the migrant-sending countries in the region [18]. In the aftermath of the Soviet Union’s collapse, the population rapidly decreased, from 5.4 million in 1989 to 3.7 million in 2022, mainly due to international labour migration [19, 20]. As a result, more than 55% of migrants are now comprised of women, and approximately 40% are children and adolescents who may be considered as/called ‘left-behind’ or ‘staying behind’, denoting that they live with caregivers while their parent(s) work abroad [18]. Existing literature suggests that left-behind children and adolescents are particularly vulnerable to mental illnesses [21–24]. Following the rise of parental out-migration in Georgia, the percentage of youth and adults with mental health disorders has increased five times (from 4.0 to 2010 to 20.0 per 100,000 in 2021). This is while in children below the age of fourteen, the prevalence increased from 0.4 to 2010 to 3.0 per 100,000 children in 2021, which is nearly an 8-fold increase [25].

The study aims to examine the emotional and behavioural difficulties of adolescents in Georgia from a public mental health perspective. To our knowledge, this is the first study using the Achenbach System of Empirically Based Assessment (ASEBA) Youth Self-Report (YSR) in Georgia. A better understanding of problem behaviours in the context of gender, parental migration and other demographic factors of adolescents in Georgia would in turn facilitate identifying mental health needs and developing more targeted interventions.

## Methods

### Study design and data

In this school-based cross-sectional study, we collected data from October 2021 to January 2022. We purposively invited 22 public schools from urban and rural areas in the Samegrelo-Zemo Svaneti region to participate in the study. All except one school principal agreed to partake in the study and three schools were closed during the data collection phase due to increased COVID-19 cases. We selected the Samegrelo-Zemo Svaneti region as it is located in the Central, North-Western part of Georgia and borders the Black Sea, Abkhazia and Russia [26]. With 10.8% of the country’s territory and 301 thousand residents, Samegrelo-Zemo Svaneti is the second-largest region in Georgia [26].

In each school, we invited all students from grades 7 to 12 (ages 12–18) to participate in our study. We arranged meetings with class tutors, asking for their assistance before and during the data collection process. The tutors assisted with arranging meetings and collecting consent forms from students and parents/caregivers. After explaining the questionnaire and the scoring method/guidelines, the data collection team (the first author and a research assistant) surveyed all students whose parents had given consent, in classrooms during/after regular school hours. Each survey took around 30–45 minutes; Data collection was anonymous; such that neither the school administration nor the tutors had access to the responses/results.

The questionnaire consisted of the Youth Self Report (YSR) of the ASEBA System supplemented with a few additional questions (listed in Appendix A1) to obtain socio-demographic information, such as the number of siblings and household members, as well as parental migration experience [27]. The YSR is a widely used child-report measure for adolescents aged 11–18 that assesses problem behaviours along two broadband scales: internalizing and externalizing, with 117 items classified as problem items or socially desirable items (listed in Appendix A2), each scored: 0 = not true, 1 = somewhat true, or 2 = very true/often true [28].

The ASEBA manual compares multicultural norm scores with borderline and clinical cut-off scores for children living in different societies [28]. For societies/countries without a norm score, such as Georgia, it is recommended to use the ASEBA Standard that equals Group 2 (societies with medium problem scores) [28, 29].

The questionnaire (ASEBA Licence N2186-10-06-20) as well as consent forms were in Georgian language and contained the contact details of the first author in case of questions/ queries. The study was approved by the Ethics Committee of the Medical Faculty of Heidelberg University N- S-160/2021 and by the Ethics Committee of the University of Georgia N-11-12401.

### Statistical analysis

Following descriptive analysis, we compared the YSR scores of the empirical syndrome items, the broadband scales between boys and girls, and the ASEBA Standard separated by gender, using two-sample t-tests.

We then, modelled the broadband scales using linear and multiple regression to identify associated variables for girls and boys, separately. The following independent variables were available for analysis: age groups (12–13, 14–15, 16–18 years); performing in any sports; number of siblings; doing household chores; having an illness (in general); the number of close friends; having school problems; living with mother, father or both parents; relationship with peers/siblings/parents; and the parents’ migration status. The variables assessing the ‘relationship with peers, siblings and parents’ asks for ‘worse, likewise or better’ relationships compared to peers. Regarding parental migration, we grouped adolescents into non-left-behind (not having a migrant parent) and left-behind (having at least one migrant parent). After bivariable analysis and collinearity check, we included all independent variables in the multiple linear regression analysis. For the statistical analysis, we used Stata version IC 15 and considered p < 0.05 as significant.

## Results

All 2,495 students between grades 7 and 12 from 18 Public schools (11 rural, 7 urban) were invited to partake in the study, of which 933 (37.9%) agreed to participate and filled out the questionnaire. The responses varied considerably across schools, ranging from 10.7 to 70.7%. Table 1 describes the detailed characteristics of participant schools.

**Table 1:** School characteristics (numbers of students, location, and participation)

School N^o^	N of Students (grade 7 to 12)	Location	Participation (%)
1	56	rural	64.3
2	72	rural	41.7
3	256	urban	70.7
4	61	rural	21.3
5	79	rural	35.4
6	112	urban	22.3
7	94	rural	39.4
8	36	rural	19.4
9	203	urban	54.2
10	148	urban	33.8
11	58	rural	46.6
12	297	urban	48.5
13	63	rural	42.9
14	89	rural	38.2
15	86	urban	68.1
16	81	rural	39.5
17	527	urban	14.6
18	177	rural	10.7
Total	2 495	-	37.4

Among the participants, 50.4% were girls, and 45.4% were boys, with the remaining 4.2% not answering the question about their gender. Most participants reported having no illness (90%), performing sports (89.3%) and stated having at least three close friends (86.7%). Team sports such as football, basketball, volleyball, and table tennis were the most reported sports activities (87.2%). Household chores such as cleaning the house and looking after siblings were done by 22.2%. Almost 90% of adolescents acknowledged having hobbies, such as reading, dancing, listening to and making music, playing cards and computer games, and making handicrafts. About a quarter admitted facing problems at school. Only 4.4% of the adolescents stated having worse relationships with their peers than other adolescents, while 67.9% claimed better relationships with their parents, and 52.2% stated having better relations with their siblings than their peers. In our sample, 76.4% of the adolescents lived with both parents, while 27.7% indicated having at least one migrant parent. Table 2 presents a detailed description of participant characteristics.

**Table 2:** Characteristics of the participants (n = 933)

Variable	*N*	%
*Gender*		
Girl	470	50.4
Boy	424	45.4
Missing	39	4.2
*Age group*		
12–13	359	38.5
14–15	336	36.0
16–18	238	25.5
*Performing any sport*		
Yes	833	89.3
No	96	10.3
Missing	4	0.4
*Number of siblings*		
0	159	17.0
1–3	399	42.8
4–5	25	2.7
≥ 6	5	0.5
Missing	345	37.0
*Doing chores*		
Yes	207	22.2
No	717	76.8
Missing	9	1.0
*Having an illness*		
Yes	64	6.9
No	840	90.0
Missing	29	3.1
*Number of close friends*		
0	24	2.6
1–3	353	37.9
≥ 4	522	55.9
Missing	34	3.6
*Having school problems*		
Yes	237	25.4
No	643	68.9
Missing	53	5.7
*Living with*		
Mother only	84	9.0
Father only	37	13.0
Both	712	76.3
Missing	100	10.7
*Relationship with peers (compared to peers)*		
Worse	41	4.4
Likewise	396	42.4
Better	416	44.6
Missing	80	8.6
*Relationship with siblings (compared to peers)*		
Worse	37	4.0
Likewise	268	28.7
Better	487	52.2
Do not have siblings	97	10.4
Missing	44	4.7
*Relationship with parents (compared to peers)*		
Worse	21	2.2
Likewise	192	20.6
Better	633	67.9
Missing	87	9.3
*Having at least one migrant parent*		
Yes	258	27.7
No	615	65.9
Missing	60	6.4

Table 3 shows the comparison of empirical syndrome scales and the broadband scales internalizing and externalizing among boys and girls within our sample and the comparison to the ASEBA Standard. The analysis revealed higher scores among girls for all syndromes except for rule-breaking behaviour, in which boys scored higher. The internalizing score was also higher among girls, while no difference in externalizing score could be observed. Adolescents who did not indicate their gender obtained similar mean scores as boys (internalizing 9.5, externalizing 10.7).

Compared to the ASEBA Normative Sample, the girls and boys in our sample obtained higher scores for all scales, except for the empirical syndromes such as ‘somatic complains’ and ‘thoughts problems’ among boys. In broadband internalizing, we observed only a minor difference among boys. Generally, differences in mean scores were noted to be larger for girls than for boys.

All scales remained within the normal range of the ASEBA standard group 2.

**Table 3:** Youth Self-Report Emotional and behavioural problems - descriptive statistics for syndrome and broadband scales

						ASEBA Normative Sample^1^			
Outcome	Girls			Boys		Girls		Boys	
**Empirically based syndromes**	N	mean (95% CI)	N	mean (95% CI)	p-value^2^	mean (normal range^3^)	p-value^4^	mean (normal range)	p-value^4^
Anxious/ Depressed	370	7.2 (6.7–7.7)	338	3.9 (3.6–4.3)	< 0.001	5.1 (< 12.0)	< 0.001	3.4 (< 9.0)	0.016
Withdrawn/ Depressed	424	5.4 (5.1–5.8)	372	4.0 (3.7–4.3)	< 0.001	3.1 (< 8.0)	< 0.001	2.7 (< 7.0)	< 0.001
Somatic Complains	384	4.7 (4.3-5.0)	362	2.2 (2.0-2.5)	< 0.001	3.4 (< 8.0)	< 0.001	2.3 (< 7.0)	0.658
Social Problems	420	4.1 (3.8–4.4)	359	3.4 (3.1–3.7)	0.002	3.2 (< 8.0)	< 0.001	2.9 (< 8.0)	0.006
Thought Problems	321	5.1 (4.6–5.7)	256	3.2 (2.8–3.6)	< 0.001	3.7 (< 10.0)	< 0.001	3.1 (< 9.0)	0.715
Attention Problems	429	6.3 (6.0-6.6)	367	5.4 (5.1–5.8)	< 0.001	4.6 (< 10.0)	< 0.001	4.9 (< 10.0)	0.014
Rule-Breaking Behaviour	409	3.8 (3.4–4.2)	347	4.7 (4.3–5.1)	0.001	3.5 (< 9.0)	0.228	3.8 (< 10.0)	< 0.001
Aggressive behaviour	424	9.2 (8.7–9.7)	352	7.3 (6.8–7.8)	< 0.001	6.5 (< 14.0)	< 0.001	6.0 (< 13.0)	< 0.001
Other problems	422	4.7 (4.5-5.0)	362	4.0 (3.8–4.3)	< 0.001	-	-	-	-
**Broadband scales**									
Internalizing	305	16.9 (15.7–18.1)	305	9.8 (9.0-10.6)	< 0.001	11.6 (< 19.0)	< 0.001	8.3 (< 14.0)	0.001
Externalizing	391	13.0 (12.2–13.9)	318	12.0 (11.2–12.8)	0.081	9.9 (< 16.0)	< 0.001	9.8 (< 17.0)	< 0.001

*Number of observations, mean score, and p-values for comparisons between girls and boys, as well as to the ASEBA Normative Sample*

^*1*^ *ASEBA Standard – multicultural normative samples group 2 adopted from the Achenbach and Rescorla (2007)*

^*2*^ *Comparison between girls and boys from the study sample*

^*3*^ *Normal range – given by the ASEBA for group 2*

^*4*^ *Comparison between ASEBA Normative Sample and the study sample*

Tables 4 and 5 present the results of the multiple regression analyses among girls and boys, respectively. Results of bivariable models are similar and shown in Appendix A4 and A5.

**Table 4:** Multiple linear regression using the broadband internalizing and externalizing scales as the outcomes for girls

	Internalizing		Externalizing	
Independent Variable	Coefficient (95% CI)	p-value	Coefficient (95% CI)	p-value
**Age group**		0.335		0.268
12–13	Ref.		Ref.	
14–15	1.61 (-2.16–5.37)		2.81 (0.05–5.57)	
16–18	-1.52 (-5.33–2.29)		0.19 (-2.71–3.09)	
**Performing any Sport**		**0.008**		0.197
Yes	Ref.		Ref.	
No	3.19 (-1.46–7.85)		-2.64 (-6.04–0.76)	
**Number of siblings**		0.740		0.329
0	Ref.		Ref.	
1–3	5.00 (-18.41–28.42)		7.92 (-4.86–20.71)	
4–5	9.85 (-14.34–34.04)		6.12 (-7.40–19.65)	
≥ 6	3.19 (-23.44–29.83)		0.22 (-16.55 -16.99)	
**Doing Chores**		0.126		0.329
Yes	0.12 (-3.30–3.55)		− 0.25 (-2.79–2.30)	
No	Ref.		Ref.	
**Having an illness**		**< 0.001**		**0.001**
Yes	Ref.		Ref.	
No	-6.17 (-12.79–0.46)		-2.85 (-6.96–1.26)	
**Number of Close Friends**		**< 0.001**		**0.015**
0	10.20 (1.85–18.56)		1.51 (-5.61–8.63)	
1–3	0.74 (-2.60–4.08)		− 0.10 (-2.63–2.43)	
≥ 4	Ref.		Ref.	
**Having School problems**		**< 0.001**		**< 0.001**
Yes	4.69 (1.30–8.07)		1.24 (-1.26–3.74)	
No	Ref.		Ref.	
**Living with**		0.075		*0.063*
Mother only	-2.90 (-7.85–2.06)		0.68 (-2.76–4.13)	
Father only	0.08 (-8.48–8.64)		− 4.45 (-10.70–1.80)	
Both parents	Ref.		Ref.	
**Relationship with peers** (compared to peers)		**< 0.001**		**0.007**
Worse	6.90 (− 0.98–14.79)		1.49 (-3.68–6.66)	
Likewise	Ref.		Ref.	
Better	-4.01 (-7.39 - -0.63)		-2.16 (-4.70–0.37)	
**Relationship with siblings** (compared to peers)		**< 0.001**		0.051
Worse	6.36 (-15.40–28.12)		9.74 (-1.70–21.17)	
Likewise	Ref.		Ref.	
Better	0.39 (-3.48–4.27)		-0.88 (-3.68–1.92)	
Do not have siblings	6.03 (-17.48–29.54)		6.71 (-6.40–19.82)	
**Relationship with parents** (compared to peers)		**< 0.001**		**< 0.001**
Worse	5.32 (-8.18–18.81)		10.41 (1.89–18.93)	
Likewise	Ref.		Ref.	
Better	-7.15 (-11.41 - -2.89)		-3.23 (-6.29 - -0.19)	
**Having at least one migrant parent**		0.445		0.107
Yes	1.72 (-1.71–5.16)		2.77 (0.18–5.37)	
No	Ref.		4.61 (1.73–7.49)	
***Const.***	21.07 ( -3.75–45.90)	0.095	9.65 (-4.61–23.91)	0.183
***Adj. R*** ^***2***^	0.3371		0.1978	

**Table 5:** Multiple linear regression using the broadband internalizing and externalizing scales as the outcomes for boys

	Internalizing		Externalizing		
Independent Variable	Coefficient (95% CI)	p-value	Coefficient (95% CI)	p-value	
**Age group**		0.549		**0.003**	
12–13	Ref.		Ref.		
14–15	-0.99 (-3.49–1.50)		1.51 (-1.29–4.32)		
16–18	0.95 (-1.85–3.75)		2.96 (-0.33–6.24)		
**Performing any Sport**		0.093		0.438	
Yes	Ref.		Ref.		
No	1.43 (-2.67–5.54)		-0.83 (-5.60–3.93)		
**Number of Siblings**		0.163		0.600	
0	Ref.		Ref.		
1–3	− 0.26 (-7.10–6.56)		0.70 (-8.33–9.72)		
4–5	4.00 (-4.72–12.72)		7.62 (-3.11–18.35)		
≥ 6	-1.84 (-11.89–8.20)		-2.75 (-14.94–9.43)		
**Doing chores**		0.393		0.780	
Yes	0.26 (-2.44–2.96)		-1.90 (-5.05 -1.25)		
No	Ref.		Ref.		
**Having an illness**		**< 0.001**		0.081	
Yes	Ref.		Ref.		
No	-4.09 (-8.31–0.13)		-2.26 (-7.23–2.72)		
**Number of Close Friends**		**< 0.001**		0.311	
0	25.91 (11.17–40.66)		-20.78 (-37.67 - -3.88)		
1–3	0.40 (-1.92–2.71)		-0.36 (-3.00–2.27)		
≥ 4	Ref.		Ref.		
**Having school problems**		**0.007**		**0.015**	
Yes	3.64 (1.05–6.23)		3.58 (0.69–6.47)		
No	Ref.		Ref.		
**Living with**		0.395		0.794	
Mother only	2.75 (-1.14–6.64)		-2.83 (-7.19–1.53)		
Father only	0.10 (-4.53–4.33)		-1.83 (-6.99–3.32)		
Both parents	Ref.		Ref.		
**Relationship with peers** (compared to peers)		**< 0.001**		0.672	
Worse	7.08 (0.01–14.15)		7.35 (-1.83–16.53)		
Likewise	Ref.		Ref.		
Better	-1.89 (-4.15–0.36)		-0.72 (-3.27–1.83)		
**Relationship with siblings** (compared to peers)		0.126		**0.024**	
Worse	-4.99 (-16.81–6.84)		-6.59 (-17.40–4.22)		
Likewise	Ref.		Ref.		
Better	-1.88 (-4.43–0.66)		-1.76 (-4.70–1.17)		
Do not have siblings	-2.50 (-9.62–4.62)		-2.36 (-11.42–6.70)		
**Relationship with parents** (compared to peers)		0.068		0.050	
Worse	-3.61 (-14.59–7.36)		3.99 (-7.21 -15.18)		
Likewise	Ref.		Ref.		
Better	-0.47 (-3.48–2.53)		-1.66 (-5.04–1.72)		
**Having at least one migrant parent**		0.932		0.449	
Yes	0.27 (-2.27–2.80)		4.61 (1.73–7.49)		
No	Ref.		Ref.		
***Constant***	15.63 (7.07–24.20)	< 0.001	13.15 (2.09–24.22)	0.020	
***Adj. R*** ^***2***^	0.3177		0.1195		

Multiple regression analysis showed associations between girls with higher internalizing scores and not performing any sports, having less than three close friends, experiencing school problems, and reporting worse relationships with peers, siblings, and parents. While having better relationships with peers and parents and not having an illness were associated with lower internalizing scores. Performing in any sport and having relationships with siblings had no association with externalizing scores.

Multiple linear regression analysis for boys revealed associations between higher internalizing scores, having less than three close friends, experiencing school problems, and having worse relationships with peers. In contrast, not having an illness and better relationships with peers were associated with lower internalizing scores. School problems and age bracket of 16–18 years was associated with higher externalizing scores. Additionally, relationships with siblings appeared to be associated with externalizing; however, better/worse relationships and not having siblings showed an association with lower externalizing scores (Table 5).

## Discussion

This study examined the emotional and behavioural problems of Georgian schoolchildren using Achenbach’s Youth Self Report. While boys expressed more rule-breaking behaviour in our study, girls displayed more problem behaviours and internalizing symptoms, indicating the gender gap. Compared to the ASEBA normative standard, Georgian adolescents in both genders showed more emotional and behavioural problems, with a gap that was even wider among girls. Importantly, factors such as the relationship with peers/siblings/parents, number of close friends and school problems might influence the results.

Our findings on internalizing problems are consistent with evidence from previous studies [2, 30–33]. For example, a comparative study of Campbell, Bann [2] from 73 countries suggested worse internalizing outcomes among girls. Remarkably, the wealthiest countries in Europe with the highest gender equality index show the most significant gender gaps [2]. Irrespective of socio-economic conditions, teenage girls perceive their health as poorer [30, 31] and express higher levels of anxiety and psychological distress [32, 34–36]. Research, including longitudinal data, demonstrates an increasing trend of emotional problems among girls [33], while a minor increase is observed among European boys [37]. Various theories have been proposed to explain this observation, mostly suggesting individual [34], biological (puberty-related hormonal changes) [38, 39], social relationships [40] and reporting differences [41]. Some academics have also suggested that shifting educational paradigms towards female education have led teenage girls to experience increased educational pressure and psychological distress [34].

Furthermore, non-differing externalizing problems among Georgian girls and boys in our study align with the American normative standard [29]; however, it contradicts the leading international literature on this issue [8, 34, 36]. For example, a study of more than 27,000 adolescents from 24 countries showed worse externalizing problems among boys [8]. Even though stressful life events seem to intensify externalizing problems in both genders, the increase is higher in boys [36]. To explain this difference, some scholars have suggested that boys are more vulnerable to self-criticism [36]. Another possible explanation is that generally, in self-reports, girls focus more on internalizing, and boys focus on externalizing problems [34]. While gender effects on internalizing tend to be universal, it seems more inconsistent with externalizing.

Similar to our results, other scholars have found that a lack of friendships and relationship difficulties with parents are associated with emotional and behavioural problems among European youths [42]. Conflicts within families were also found to increase the risk of developing mental health disorders among youth in Lithuania [43]. In contrast, close ties and relationships with one’s family members correlates with better mental health and reduced risky behaviour during adolescence [42, 44]. Moreover, close friendships may even prevent and mitigate aggression and antisocial behaviour among adolescent boys [45]. Given that boys in Georgia display higher rule-breaking behaviour, developing friendships and social connections are believed to bear an important impact on the mitigation of this behaviour in this population.

Consistent with our study’s findings, other scholars suggest an association between school environment and psychological distress among adolescents [34, 46–48]. School stress is mainly discussed in the context of increased schoolwork pressure, conflicts with schoolteachers, and peer bullying [46]. School problems may increase psychosomatic complaints, anxiety, and psychological distress [34] and even lead adolescents to ‘school burnout’ [47]. Girls seem more responsible for performing well at school, which may cause an imbalance between increased pressure and available resources (e.g. needed support), eventually leading to stress and anxiety [34, 49]. In contrast, a multi-country study by Bradshaw, Martorano [48] suggests that a supportive school environment and good relationships with classmates are crucial determinants of adolescents’ well-being and happiness. Similar to youths from other countries, adolescents in our study could experience problems at school because of schoolwork pressure and conflicts with teachers and classmates. School factors could also explain more significant internalizing problems that was observed among teenage girls in our sample.

In general, the gender gap is described as a cross-cultural phenomenon [3, 16, 50, 51]. In addition to the socio-economic conditions, its size seems to be affected by the area of residence (rural/urban) [50], migration background and ethnicity [3, 16, 50, 51]. Considering the migration profile of Georgia, parental migration was another factor we investigated in this study. We found that adolescents with or without parental migration face similar emotional and behavioural difficulties. Our findings only partially reflect evidence from previous studies in this field from Georgia, where some scholars argue that children of migrant parents are vulnerable to poor mental health [52]; while others suggest that parents’ migration is associated with children’s improved mental health and well-being [53–55]. Worse mental health outcomes are indicated among left-behind children in other Eastern European countries [56–58] and internationally [21, 24, 59–62]. Several factors could explain this inconsistency. Our study involves adolescents above 11, while most existing studies include children of all ages (0–18). Younger children could be more vulnerable to migratory separation. Informants, other than adolescents may evaluate parental migration impact more negatively. As Lee, Park [63] suggested, self-reported emotional problems of adolescents are more reliable than caregiver reports. Other factors, such as closeness with a migrant parent, good relationships with family members and social interactions, may mitigate the adverse effects of parental absence and, consequently, explain non-differing problem behaviours among left-behind and non-left-behind adolescents in Georgia and elsewhere.

Less than 40% of targeted youths participated in our study. We believe the possible reasons are threefold: (1) There might have been a COVID-19 pandemic-related fear among the public, as many students were absent in the classroom; (2) In Georgia, completing informed consent forms as part of an official request to participate in a research project is rare - it could be seen as “dangerous”; (3) The YSR questionnaire was anonymous, however, many adolescents and their families might still find the subject too sensitive. Our study is the first attempt to encourage young people, their families, schools and communities to collaborate with scientists on similar research projects in the future.

Several elements in our research provide a different perspective/viewpoint and more insightful conclusions to the body of existing literature. We investigated adolescents’ emotional and behavioural problems in Georgia, addressing a major research gap in Eastern Europe. Instead of adults, we asked adolescents to report on their problem behaviours, because they are indispensable informants of their mental health. We applied ASEBA tool, the YSR, which is identified among the 11 most reliable measures in child psychopathology [6] and is increasingly used in research worldwide [8, 9, 28, 29]. The YSR had only been applied in clinical practice in Georgia up until now. Finally, we considered parental migration status, which is a critical sociodemographic factor for Georgia.

This study had several limitations. We collected data during the COVID-19 pandemic when the government of Georgia allowed schools to re-open after three semesters of school closure. Schools were given the autonomy to decide between online or in-classroom teaching. Our initial plan of random sample selection was not possible. The COVID-19 pandemic and school closures could have also influenced the results. All included schools were in the Samegrelo-Zemo Svaneti region; thus, our findings are not representative of other areas or the entire population of Georgia. As stated above, the overall response rate in our study is low and varies across schools. Responses of adolescents who missed school on the day of data collection could differ. Not all adolescents who participated in the study answered all questions. We excluded all observations with missing YSR problem items from the analysis. These study findings are based on information that was self-reported by adolescents. Including other informants such as teachers, parents or caregivers and comparing results could provide a broader picture/more insights. Lastly, the cross-sectional design of the study does not allow us to conclude on possible causes of emotional and behavioural problems of adolescents in Georgia.

## Conclusion

The findings of this study highlight the need to address the emotional and behavioural difficulties that Georgian adolescents face, particularly internalizing problems in girls and rule-breaking behaviour in boys. Our results reflect the increasing trend of child and adolescent mental health disorders in Georgia. Relationships within family, social connections and school environment appear as important contributors. Our findings indicate that social support interventions may protect adolescents from experiencing emotional and behavioural difficulties in Georgia.

Finally, we emphasize the necessity of raising public awareness for child and adolescent mental health issues. It is essential that adolescents, schools and parents understand the potential benefits of participating in similar research projects to ours. We further encourage collaborations between state and non-governmental organizations, child and adolescent mental health professionals, as well as global health scientists in developing/promoting mental health interventions involving adolescents, their families, and communities in Georgia.

## Electronic supplementary material

Below is the link to the electronic supplementary material.

Supplementary Material 1

## Data Availability

Collected data is stored in a password-protected hard drive at Heidelberg Institute of Global Health. Datasets generated and/or analysed during the current study are available from the corresponding author on reasonable request.

## List of Abbreviations

YSR: Youth Self-Report
ASEBA: The Achenbach System of Empirically Based Assessment
SDG: Sustainable Development Goals
UNICEF: The United Nations Children’s Fund
COVID-19: Coronavirus Disease 2019
