# Supplementary material for: The gender gap in adolescents’ emotional and behavioural problems in Georgia: a cross-sectional study using Achenbach’s Youth Self Report
Source: Child Adolesc Psychiatry Ment Health. 2023 Mar 30;17:44. doi: 10.1186/s13034-023-00592-0 (PMC10061395; doi:10.1186/s13034-023-00592-0)
Supplement: Supplementary file 1 — Supplementary Material 1 [file 13034_2023_592_MOESM1_ESM.docx]

**Appendix**

**A1: Additional Questionnaire**

1. **How many brothers and how many sisters do you have?**
   □ No brothers and no sisters
   □ Brothers (add number) ____
   □ Sisters (add number) ____
2. **Whom do you live with? (Cross all that is true for you)**

□ Mother
□ Father

□ Grandmother

□ Grandfather
□ Relatives

□ Other (specify)________________

1. **Which of the following statement describes your situation best?**

□ My mother was abroad but returned during the Covid-19 pandemic
□ My father was abroad but returned during the Covid-19 pandemic

□ Both of my parents were abroad, and both returned during the Covid-19 pandemic

□ None of my parents were abroad

□ One (or both) of my parents remains abroad

**Continue only if a parent (one or both) is currently abroad**

1. **How long has your parent(s) been living abroad?**

**Mother**
□ Less than a year
□ More than a year

**Father**

□ Less than a year
□ More than a year

1. **How often does your parent(s) visit home?**

□ Once in several months

□ Once a year

□ Once in several years

1. **How often do you communicate with your parents over a distance (Skype, Messenger, Viber, or other programs)?**
   □ Daily
   □ Weekly
   □ Monthly
   □ Less than a month

**A2:** Youth Self Report (YSR) empirical syndrome scales, items in each scale, total number, mean and standard deviation, separate for girls and boys.

| **YSR Empirical Syndrome Scale** | | **Girls** | | | **Boys** | | | **Total** | | |
| --- | --- | --- | --- | --- | --- | --- | --- | --- | --- | --- |
| *Item* | | *N* | *mean* | *SD* | *N* | *mean* | *SD* | *N* | *mean* | *SD* |
| 1. **Anxious/Depressed** | |  | | |  | | |  | | |
| 14. | Cries a lot | 462 | 0.63 | 0.82 | 412 | 0.12 | 0.70 | 874 | 0.40 | 0.80 |
| 29. | Fears | 425 | 0.81 | 0.80 | 391 | 0.55 | 0.71 | 816 | 0.69 | 0.77 |
| 30. | Fears school | 461 | 0.09 | 0.32 | 408 | 0.06 | 0.50 | 869 | 0.07 | 0.42 |
| 31. | Fears doing bad | 463 | 0.48 | 0.67 | 406 | 0.35 | 0.72 | 869 | 0.42 | 0.70 |
| 32. | Must be perfect | 447 | 0.56 | 0.69 | 394 | 0.39 | 0.71 | 841 | 0.48 | 0.70 |
| 33. | Feels unloved | 459 | 0.53 | 0.69 | 407 | 0.29 | 0.70 | 866 | 0.42 | 0.70 |
| 35. | Feels worthless | 460 | 0.50 | 0.80 | 411 | 0.25 | 0.50 | 871 | 0.38 | 0.69 |
| 45. | Nervous, tense | 460 | 0.80 | 0.74 | 408 | 0.53 | 0.79 | 868 | 0.67 | 0.77 |
| 50. | Fearful, anxious | 463 | 0.62 | 0.74 | 408 | 0.23 | 0.66 | 871 | 0.44 | 0.73 |
| 52. | Feels too guilty | 463 | 0.41 | 0.62 | 410 | 0.29 | 0.67 | 873 | 0.35 | 0.64 |
| 71. | Self-conscious | 458 | 1.00 | 0.71 | 402 | 0.79 | 0.73 | 860 | 0.90 | 0.73 |
| 91. | Talks or thinks of suicide | 456 | 0.20 | 0.50 | 401 | 0.07 | 0.30 | 857 | 0.13 | 0.43 |
| 112. | Worries | 430 | 0.54 | 0.70 | 385 | 0.25 | 0.54 | 815 | 0.40 | 0.64 |
| 1. **Withdraw/Depressed** | |  | | |  | | |  | | |
| 5 | Enjoys little | 458 | 0.78 | 0.78 | 405 | 0.81 | 1.00 | 863 | 0.79 | 0.89 |
| 42 | Rather be alone | 459 | 0.83 | 0.92 | 407 | 0.52 | 0.97 | 866 | 0.68 | 0.95 |
| 65 | Won’t talk | 456 | 0.42 | 0.61 | 402 | 0.40 | 0.63 | 858 | 0.41 | 0.62 |
| 69 | Secretive | 461 | 0.92 | 0.76 | 400 | 0.80 | 1.08 | 861 | 0.86 | 0.93 |
| 75 | Shy, timid | 456 | 0.72 | 0.74 | 398 | 0.58 | 0.66 | 854 | 0.65 | 0.71 |
| 102 | Lacks energy | 457 | 0.61 | 0.69 | 403 | 0.30 | 0.69 | 860 | 0.46 | 0.71 |
| 103 | Sad | 456 | 0.45 | 0.67 | 402 | 0.18 | 0.61 | 858 | 0.33 | 0.66 |
| 111 | Withdrawn | 450 | 0.63 | 0.98 | 397 | 0.48 | 0.79 | 847 | 0.56 | 0.90 |
| 1. **Somatic Complaints** | |  | | |  | | |  | | |
| 47. | Nightmares | 463 | 0.62 | 0.70 | 409 | 0.40 | 0.72 | 872 | 0.51 | 0.72 |
| 51. | Feels dizzy | 464 | 0.50 | 0.71 | 406 | 0.18 | 0.61 | 870 | 0.35 | 0.69 |
| 54. | Tired | 458 | 0.82 | 0.75 | 409 | 0.46 | 0.78 | 867 | 0.65 | 0.79 |
| 56a. | Aches, pains | 420 | 0.32 | 0.80 | 386 | 0.16 | 0.45 | 806 | 0.24 | 0.66 |
| 56b. | Headaches | 441 | 0.84 | 0.88 | 391 | 0.36 | 0.56 | 832 | 0.62 | 0.79 |
| 56c. | Nausea | 431 | 0.35 | 0.60 | 389 | 0.17 | 0.44 | 820 | 0.26 | 0.54 |
| 56d. | Eye problems | 413 | 0.17 | 0.46 | 379 | 0.13 | 0.58 | 792 | 0.15 | 0.52 |
| 56e. | Skin problems | 445 | 0.59 | 0.72 | 396 | 0.25 | 0.52 | 841 | 0.43 | 0.65 |
| 56f. | Stomach-aches | 452 | 0.65 | 0.64 | 397 | 0.27 | 0.49 | 849 | 0.47 | 0.60 |
| 56g. | Vomiting | 442 | 0.11 | 0.52 | 395 | 0.66 | 0.30 | 837 | 0.09 | 0.43 |
| 1. **Social problems** | |  | | |  | | |  | | |
| 11. | Dependent | 455 | 0.52 | 0.60 | 407 | 0.50 | 0.73 | 862 | 0.51 | 0.67 |
| 12. | Lonely | 461 | 0.59 | 0.75 | 410 | 0.35 | 0.63 | 871 | 0.48 | 0.70 |
| 25. | Doesn’t get along with other kids | 464 | 0.48 | 0.78 | 410 | 0.43 | 0.79 | 874 | 0.46 | 0.78 |
| 27. | Jealous | 463 | 0.20 | 0.46 | 412 | 0.14 | 0.70 | 875 | 0.17 | 0.58 |
| 34. | Others out to get him/her | 458 | 0.19 | 0.49 | 406 | 0.20 | 0.63 | 864 | 0.19 | 0.56 |
| 36. | Accident-prone | 461 | 0.57 | 0.68 | 411 | 0.71 | 0.91 | 872 | 0.64 | 0.80 |
| 38. | Gets teased | 462 | 0.30 | 0.58 | 411 | 0.22 | 0.76 | 873 | 0.26 | 0.67 |
| 48. | Not liked | 460 | 0.39 | 0.61 | 405 | 0.28 | 0.68 | 865 | 0.34 | 0.65 |
| 62. | Clumsy | 453 | 0.40 | 0.28 | 400 | 0.33 | 0.54 | 853 | 0.36 | 0.58 |
| 64. | Prefers younger kids | 455 | 0.34 | 0.56 | 401 | 0.34 | 0.83 | 856 | 0.34 | 0.70 |
| 79. | Speech problems | 451 | 0.16 | 0.44 | 387 | 0.14 | 0.41 | 838 | 0.15 | 0.43 |
| 1. **Thought problems** | |  | | |  | | |  | | |
| 9. | Can’t get mind off thoughts | 424 | 0.89 | 0.87 | 375 | 0.64 | 0.92 | 799 | 0.77 | 0.91 |
| 18. | Harms self | 464 | 0.17 | 0.46 | 407 | 0.59 | 0.30 | 871 | 0.12 | 0.40 |
| 40. | Hears things | 449 | 0.27 | 0.72 | 400 | 0.16 | 0.63 | 849 | 0.22 | 0.68 |
| 46. | Twitching | 450 | 0.35 | 0.64 | 393 | 0.15 | 0.60 | 843 | 0.25 | 0.63 |
| 58. | Picks skin | 436 | 0.35 | 0.61 | 388 | 0.19 | 0.46 | 824 | 0.28 | 0.55 |
| 66. | Repeats acts | 426 | 0.36 | 0.65 | 384 | 0.23 | 0.52 | 810 | 0.30 | 0.60 |
| 70. | Sees things | 437 | 0.20 | 0.51 | 391 | 0.17 | 0.77 | 828 | 0.18 | 0.65 |
| 76. | Sleeps less | 458 | 0.74 | 0.83 | 398 | 0.66 | 0.86 | 856 | 0.70 | 0.85 |
| 83. | Stores things | 450 | 0.73 | 0.80 | 393 | 0.49 | 0.71 | 843 | 0.62 | 0.77 |
| 84. | Strange behaviour | 453 | 0.40 | 0.67 | 392 | 0.25 | 0.53 | 845 | 0.33 | 0.61 |
| 85. | Strange ideas | 400 | 0.46 | 0.71 | 323 | 0.30 | 0.61 | 723 | 0.39 | 0.67 |
| 100. | Trouble sleeping | 444 | 0.45 | 0.71 | 388 | 0.21 | 0.53 | 832 | 0.34 | 0.64 |
| 1. **Attention Problems** | |  | | |  | | |  | | |
| 1. | Acts Young | 460 | 0.56 | 0.59 | 401 | 0.46 | 0.67 | 861 | 0.51 | 0.58 |
| 4. | Fails to finish things | 456 | 0.63 | 0.69 | 404 | 0.51 | 0.65 | 860 | 0.57 | 0.67 |
| 8. | Can’t concentrate | 459 | 0.72 | 0.67 | 411 | 0.61 | 0.66 | 870 | 0.66 | 0.66 |
| 10. | Can’t sit still | 460 | 0.51 | 0.66 | 410 | 0.62 | 0.68 | 870 | 0.56 | 0.67 |
| 13. | Confused | 460 | 0.56 | 0.73 | 410 | 0.28 | 0.67 | 870 | 0.42 | 0.72 |
| 17. | Daydreams | 463 | 1.38 | 0.67 | 410 | 1.15 | 0.91 | 873 | 1.28 | 0.80 |
| 41. | Impulsive | 459 | 0.68 | 0.64 | 409 | 0.60 | 0.85 | 868 | 0.64 | 0.75 |
| 61. | Poor Schoolwork | 460 | 0.57 | 0.74 | 402 | 0.65 | 0.67 | 862 | 0.61 | 0.71 |
| 78. | Inattentive | 458 | 0.66 | 0.70 | 400 | 0.61 | 0.78 | 858 | 0.64 | 0.74 |
| 1. **Rule-breaking behaviour** | |  | | |  | | |  | | |
| 2. | Drinks alcohol | 455 | 0.23 | 0.51 | 397 | 0.24 | 0.51 | 852 | 0.24 | 0.52 |
| 26. | Lacks guilt | 459 | 0.57 | 0.86 | 408 | 0.59 | 0.75 | 867 | 0.58 | 0.81 |
| 28. | Breaks rules | 458 | 0.29 | 0.51 | 408 | 0.42 | 0.81 | 866 | 0.35 | 0.67 |
| 39. | Bad friends | 460 | 0.37 | 0.60 | 409 | 0.45 | 0.64 | 869 | 0.40 | 0.62 |
| 43. | Lies, cheats | 461 | 0.43 | 0.61 | 409 | 0.38 | 0.68 | 870 | 0.41 | 0.64 |
| 63. | Prefers older kids | 453 | 0.60 | 0.69 | 401 | 0.60 | 0.82 | 854 | 0.60 | 0.76 |
| 67. | Runs away | 462 | 0.11 | 0.37 | 401 | 0.05 | 0.26 | 863 | 0.08 | 0.32 |
| 72. | Sets fires | 458 | 0.16 | 0.45 | 401 | 0.24 | 0.54 | 859 | 0.20 | 0.50 |
| 81. | Steals at home | 457 | 0.04 | 0.25 | 400 | 0.07 | 0.31 | 857 | 0.05 | 0.25 |
| 82. | Steals outside home | 454 | 0.04 | 0.23 | 391 | 0.06 | 0.29 | 845 | 0.05 | 0.27 |
| 90. | Swears | 460 | 0.43 | 0.74 | 403 | 0.62 | 0.69 | 863 | 0.52 | 0.72 |
| 96. | Thinks of sex too much | 454 | 0.22 | 0.74 | 398 | 0.76 | 1.34 | 852 | 0.47 | 1.10 |
| 99. | Uses tobacco | 458 | 0.06 | 0.28 | 404 | 0.11 | 0.58 | 862 | 0.08 | 0.45 |
| 101. | Truant | 458 | 0.24 | 0.66 | 403 | 0.24 | 0.50 | 861 | 0.24 | 0.59 |
| 105. | Uses drags | 446 | 0.05 | 0.28 | 392 | 0.04 | 0.25 | 838 | 0.05 | 0.27 |
| 1. **Aggressive behaviour** | |  | | |  | | |  | | |
| 3. | Argues a lot | 461 | 0.76 | 0.69 | 410 | 0.71 | 0.88 | 871 | 0.74 | 0.78 |
| 16. | Mean to others | 460 | 0.17 | 0.45 | 407 | 0.15 | 0.39 | 867 | 0.16 | 0.42 |
| 19. | Demands attention | 463 | 0.58 | 0.67 | 411 | 0.53 | 0.73 | 874 | 0.55 | 0.70 |
| 20. | Destroys own things | 463 | 0.19 | 0.48 | 412 | 0.14 | 0.58 | 875 | 0.17 | 0.53 |
| 21. | Destroys others’ things | 462 | 0.07 | 0.29 | 409 | 0.93 | 0.67 | 871 | 0.08 | 0.50 |
| 22. | Disobedient at home | 458 | 0.34 | 0.57 | 405 | 0.28 | 0.51 | 863 | 0.31 | 0.54 |
| 23. | Disobedient at school | 453 | 0.23 | 0.51 | 403 | 0.36 | 0.71 | 856 | 0.29 | 0.61 |
| 37. | Gets in fights | 463 | 0.48 | 0.65 | 404 | 0.66 | 0.90 | 867 | 0.56 | 0.78 |
| 57. | Attacks people | 458 | 0.09 | 0.33 | 401 | 0.10 | 0.37 | 859 | 0.09 | 0.35 |
| 68. | Screams a lot | 461 | 0.45 | 0.63 | 400 | 0.28 | 0.52 | 861 | 0.37 | 0.59 |
| 86. | Stubborn, sullen | 461 | 1.37 | 0.77 | 404 | 0.96 | 0.83 | 865 | 1.18 | 0.83 |
| 87. | Mood changes | 459 | 1.49 | 0.75 | 401 | 1.04 | 0.84 | 860 | 1.28 | 0.83 |
| 89. | Suspicious | 460 | 1.15 | 0.88 | 404 | 0.61 | 0.74 | 864 | 0.90 | 0.86 |
| 94. | Teases a lot | 459 | 0.12 | 0.54 | 404 | 0.16 | 0.41 | 863 | 0.14 | 0.49 |
| 95. | Temper | 461 | 1.09 | 0.79 | 403 | 0.87 | 0.80 | 864 | 0.99 | 0.80 |
| 97. | Threatens others | 458 | 0.10 | 0.36 | 401 | 0.10 | 0.37 | 859 | 0.10 | 0.36 |
| 104. | Loud | 457 | 0.49 | 0.80 | 402 | 0.50 | 0.77 | 859 | 0.49 | 0.78 |
| **Other problems** | |  | | |  | | |  | | |
| 7. | Brags | 462 | 0.21 | 0.44 | 410 | 0.29 | 0.53 | 872 | 0.25 | 0.49 |
| 24. | Doesn’t eat well | 459 | 0.79 | 0.86 | 409 | 0.67 | 0.84 | 868 | 0.73 | 0.85 |
| 44. | Bites nails | 463 | 0.46 | 0.69 | 410 | 0.55 | 0.82 | 873 | 0.50 | 0.75 |
| 53. | Overeating | 464 | 0.73 | 0.83 | 410 | 0.75 | 0.94 | 874 | 0.73 | 0.88 |
| 55. | Overweight | 462 | 0.34 | 0.73 | 404 | 0.25 | 0.68 | 866 | 0.30 | 0.71 |
| 56h. | Other physical problems | 248 | 0.18 | 0.73 | 301 | 0.08 | 0.34 | 549 | 0.13 | 0.55 |
| 74. | Shows off | 454 | 0.70 | 0.73 | 397 | 0.37 | 0.61 | 851 | 0.55 | 0.70 |
| 77. | Sleeps more | 443 | 0.47 | 0.66 | 385 | 0.41 | 0.65 | 828 | 0.44 | 0.66 |
| 93. | Talks too much | 461 | 1.10 | 0.81 | 403 | 0.87 | 0.70 | 864 | 0.99 | 0.78 |
| 110. | Wishes to be the opposite sex | 456 | 0.31 | 0.72 | 399 | 0.15 | 0.73 | 855 | 0.23 | 0.73 |

**A3**: Youth Self Report (YSR) broad-band scale scores, total problem score, computation

| **Broad-band Scale Syndrome Computation** | | |
| --- | --- | --- |
| **Internalizing (a)** | Syndrome I (Anxious/depressed)  Syndrome II (Withdrawn/depressed)  Syndrome III (Somatic Complains) | a= scale I + II+ III |
| **Externalizing (b)** | Syndrome VII (Rule-breaking behaviour)  Syndrome VIII (Aggressive behaviour) | b= scale VII + VIII |
| **Other (c)** | Syndrome IV (Social problems)  Syndrome V (Thought problems)  Syndrome VI (Attention Problems)  Other problems | c= scale IV + V+ VI + other problems |
| **Total problem score:** | Internalizing  Externalizing  Other | Total = (a)+(b)+(c) |

*ASEBA norms – Achenbach System of Empirically Based Assessment norms: Youth Self Report (YSR) scales – computation. Adopted from the Achenbach and Rescorla (2001)

**A4:** Bivariable linear regression using the broadband internalizing and externalizing scales as the outcomes for girls

|  | **Internalizing** | | **Externalizing** | |
| --- | --- | --- | --- | --- |
| **Independent Variable** | **Coefficient**  **(constant*)** | **p-value** | **Coefficient**  **(constant*)** | **p-value** |
| **Age group** |  |  |  |  |
| 12-13 | Ref. (15.96) |  | Ref. (12.31) |  |
| 14-15 | 2.08 | 0.140 | 1.58 | 0.109 |
| 16-18 | 1.02 | 0.510 | 0.55 | 0.620 |
| **Performing any Sport** |  |  |  |  |
| Yes | Ref. (16.31) |  | Ref. (12.84) |  |
| No | 4.79 | **0.008** | 1.68 | 0.197 |
| **Number of siblings** |  |  |  |  |
| 0 | Ref. (17.88) |  | Ref. (13.48) |  |
| 1-3 | - 0.98 | 0.615 | 0.18 | 0.896 |
| 4-5 | 1.32 | 0.730 | - 2.79 | 0.297 |
| ≥6 | - 6.88 | 0.381 | - 8.98 | 0.150 |
| **Doing chores** |  |  |  |  |
| Yes | 2.07 |  | 0.93 |  |
| No | Ref. (16.33) | 0.126 | Ref. (12.75) | 0.329 |
| **Having an illness** |  |  |  |  |
| Yes | Ref. (27.35) |  | Ref. (17.68) |  |
| No | - 11.26 | **<0.001** | - 5.19 | **0.001** |
| **Number of close Friends** |  |  |  |  |
| 0 | 14.11 | **<0.001** | 6.046 | **0.021** |
| 1-3 | 3.44 | 0.005 | 1.87 | **0.032** |
| ≥4 | Ref. (14.71) |  | Ref. (11.77) |  |
| **Having school problems** |  |  |  |  |
| Yes | 7.14 | **<0.001** | 3.90 | **<0.001** |
| No | Ref. (14.59) |  | Ref. (11.58) |  |
| **Living with** |  |  |  |  |
| Mother only | 2.51 | 0.226 | 2.69 | 0.064 |
| Father only | - 5.60 | 0.067 | - 2.87 | 0.189 |
| Both parents | Ref. (16.75) |  | Ref. (12.87) |  |
| **Relationship with peers**  (compared to peers) |  |  |  |  |
| Worse | 8.41 | **0.002** | 3.82 | **0.037** |
| Likewise | Ref. (19.15) |  | Ref. (13.51) |  |
| Better | - 4.89 | **<0.001** | - 1.57 | 0.078 |
| **Relationship with siblings**  (compared to peers) |  |  |  |  |
| Worse | 3.89 | 0.249 | 2.65 | 0.226 |
| Likewise | Ref (17.66) |  | Ref (14.00) |  |
| Better | - 1.55 | 0.261 | - 1.98 | **0.043** |
| Do not have siblings | 0.13 | 0.954 | - 0.57 | 0.706 |
| **Relationship with parents** (compared to peers) |  |  |  |  |
| Worse | 6.80 | 0.082 | 3.30 | 0.224 |
| Likewise | Ref. (22.91) |  | Ref. (16.5) |  |
| Better | - 8.16 | **<0.001** | - 4.97 | **<0.001** |
| **Having at least one migrant parent** |  |  |  |  |
| Yes | - 1.02 | 0.445 | 1.47 | 0.107 |
| No | Ref. (17.23) |  | Ref. (12.46) |  |
| **Const.** |  |  |  |  |
| **Adj. R^2^** |  |  |  |  |

*P-values of constants <0.001

**A5:** Bivariable linear regression using the broadband internalizing and externalizing scales as the outcomes for boys

|  | **Internalizing** | | **Externalizing** | |
| --- | --- | --- | --- | --- |
| **Independent Variable** | **Coefficient**  **(constant*)** | **p-value** | **Coefficient**  **(constant*)** | **p-value** |
| **Age group** |  |  |  |  |
| 12-13 | Ref. (10.35) |  | Ref. (10.10) |  |
| 14-15 | - 1.07 | 0.274 | 2.76 | 0.005 |
| 16-18 | - 0.51 | 0.623 | 3.30 | **0.002** |
| **Performing any Sport** |  |  |  |  |
| Yes | Ref. (9.63) |  | Ref. (11.86) |  |
| No | 2.82 | 0.093 | 1.22 | 0.438 |
| **Number of Siblings** |  |  |  |  |
| 0 | Ref. (8.32) |  | Ref. (11.90) |  |
| 1-3 | 2.50 | **0.030** | - 0.16 | 0.891 |
| 4-5 | 2.35 | 0.443 | 3.77 | 0.249 |
| ≥6 | 4.35 | 0.303 | - 2.56 | 0.570 |
| **Doing chores** |  |  |  |  |
| Yes | 0.95 | 0.393 | - 0.32 | 0.780 |
| No | Ref. (9.66) |  | Ref. (12.06) |  |
| **Having an illness** |  |  |  |  |
| Yes | Ref. (15.94) |  | Ref. (14.94) |  |
| No | - 6.55 | **<0.001** | - 3.21 | 0.081 |
| **Number of close Friends** |  |  |  |  |
| 0 | 14.70 | **<0.001** | 5.14 | 0.137 |
| 1-3 | 2.31 | **0.013** | 0.46 | 0.650 |
| ≥4 | Ref. (8.90) |  | Ref. (11.86) |  |
| **Having school problems** |  |  |  |  |
| Yes | 2.80 | **0.007** | 2.58 | **0.015** |
| No | Ref. (9.26) |  | Ref. (11.34) |  |
| **Living with** |  |  |  |  |
| Mother only | 2.04 | 0.192 | 0.40 | 0.794 |
| Father only | 0.95 | 0.621 | 0.33 | 0.867 |
| Both parents | Ref. (9.80) |  | Ref. (11.87) |  |
| **Relationship with peers**  (compared to peers) |  |  |  |  |
| Worse | 8.12 | **<0.001** | - 0.60 | 0.804 |
| Likewise | Ref. (9.96) |  | Ref. (12.42) |  |
| Better | - 1.10 | 0.209 | - 0.83 | 0.375 |
| **Relationship with siblings**  (compared to peers) |  |  |  |  |
| Worse | 4.71 | 0.078 | 4.60 | 0.055 |
| Likewise | Ref. (10.29) |  | Ref. (12.67) |  |
| Better | - 0.90 | 0.349 | - 1.39 | 0.150 |
| Do not have siblings | - 1.50 | 0.281 | - 2.52 | 0.092 |
| **Relationship with parents**  (compared to peers) |  |  |  |  |
| Worse | 2.02 | 0.518 | - 0.69 | 0.820 |
| Likewise | Ref. (11.31) |  | Ref. (13.83) |  |
| Better | - 2.11 | **0.048** | **- 2.61** | **0.016** |
| **Having at least one migrant parent** |  |  |  |  |
| Yes | 0.08 |  | 0.71 | 0.449 |
| No | Ref. (9.93) | 0.932 | Ref. (11.65) |  |
| **Constant** |  |  |  |  |
| **Adj. R^2^** |  |  |  |  |

*P-values of constants <0.001
